# The heat shock protein 90 of *Toxoplasma gondii* is essential for invasion of host cells and tachyzoite growth

**DOI:** 10.1051/parasite/2017023

**Published:** 2017-06-19

**Authors:** Hongchao Sun, Xunhui Zhuo, Xianfeng Zhao, Yi Yang, Xueqiu Chen, Chaoqun Yao, Aifang Du

**Affiliations:** 1 Institute of Preventive Veterinary Medicine & Zhejiang Provincial Key Laboratory of Preventive Veterinary Medicine, College of Animal Sciences, Zhejiang Provincial Key Laboratory of Preventive Veterinary Medicine, Zhejiang University Hangzhou 310058 PR China; 2 Shenzhen Entry-exit Inspection and Quarantine Bureau Shenzhen 518045 PR China; 3 Department of Biomedical Sciences and One Health Center for Zoonoses and Tropical Veterinary Medicine, Ross University School of Veterinary Medicine P.O. Box 334 Basseterre, St. Kitts West Indies

**Keywords:** *Toxoplasma gondii*, Heat shock protein 90, Invasion, Differentiation, Replication

## Abstract

*Toxoplasma gondii* is an obligate intracellular apicomplexan parasite that infects almost all warm-blooded vertebrates. Heat shock proteins (HSP) regulate key signal transduction events in many organisms, and heat shock protein 90 (Hsp90) plays an important role in growth, development, and virulence in several parasitic protozoa. Here, we discovered increased transcription of the *Hsp90* gene under conditions for bradyzoite differentiation, i.e. alkaline and heat shock conditions *in vitro*, suggesting that Hsp90 may be connected with bradyzoite development in *T. gondii*. A knockout of the *Tg*Hsp90 strain (*ΔHsp90*) and a complementation strain were constructed. The *Tg*Hsp90 knockout cells were found to be defective in host-cell invasion, were not able to proliferate *in vitro* in Vero cells, and did not show long-time survival in mice *in vivo.* These inabilities of the knockout parasites were restored upon complementation of *Tg*Hsp90. These data unequivocally show that *Tg*Hsp90 contributes to bradyzoite development, and to invasion and replication of *T. gondii* in host cells.

## Abstract (fr)

*Toxoplasma gondii* est un Apicomplexa parasite intracellulaire obligatoire qui infecte presque tous les vertébrés à sang chaud. Les protéines de choc thermique régulent les événements principaux de transduction du signal chez de nombreux organismes et la protéine de choc thermique 90 (Hsp90) joue un rôle important dans la croissance, le développement et la virulence chez plusieurs protozoaires parasites. Dans ce travail nous avons découvert une augmentation de la transcription du gène de Hsp90 dans les conditions de différenciation des bradyzoïtes et de chocs alcalins et thermiques *in vitro*, ce qui suggère que Hsp90 est lié au développement des bradyzoïtes chez *T. gondii*. Un knock-out de la souche *Tg*Hsp90 (*ΔHsp90*) et une souche de complémentation ont été construits. Les cellules knock-out *Tg*Hsp90 se sont révélées défectueuses dans l’invasion des cellules hôtes, n’ont pas pu proliférer *in vitro* dans des cellules Vero et n’ont pas montré de survie de longue durée chez la souris *in vivo*. Ces défauts des parasites knock-out ont été restaurés lors de la complémentation de *Tg*Hsp90. Ces données montrent sans équivoque que *Tg*Hsp90 contribue au développement des bradyzoïtes, et à l’invasion et à la réplication de *T. gondii* dans les cellules hôtes.

## Introduction

*Toxoplasma gondii* is a protozoan parasite of medical and veterinary importance. It causes toxoplasmosis in almost all homoeothermic animals, including humans, leading to potentially severe congenital infections and life-threatening conditions in the developing fetus or newborn [17, 31]. There are three infective stages of *T. gondii* including the rapidly replicating tachyzoite, the slow-replicating bradyzoite, and the sporozoite-containing oocyst. The first two are asexual replication stages in the intermediate host of most, if not all, warm-blood animals including humans. The latter is the result of the sexual replication that occurs in the definitive hosts, i.e. cats and other felids [23, 41].

*Humans become infected by eating tissue cysts in undercooked meat, consuming oocyst-contaminated food or water, or accidentally ingesting oocysts in cat feces* [26]. *The ability of T. gondii to cycle between the tachyzoite and bradyzoite stages in intermediate hosts is the key to its survival and a major factor in the pathogenesis of toxoplasmosis* [12]*.* Although the cycle between tachyzoite and bradyzoite is critical to parasite survival, the underlying molecular mechanisms remain largely unknown to date [39, 46, 54].

Heat shock protein (HSP) families are evolutionarily conserved in many organisms throughout various taxa [30] and were first discovered in *Drosophila* in 1962 [25]. They are important molecular chaperones for maintaining cellular functions to prevent proteins from misfolding and aggregation in crowded surroundings [19, 50]. Their expression levels increase dramatically when the cells are cultured under stress conditions [28] such as heat shock, alkaline treatment, and some chemical reagents. In the past several years, a number of research groups have shown that the HSPs are not only involved in protein modification and folding but also participate in many signaling pathways [40]. Furthermore, HSPs affect the immune system, such as binding antigen during antigen processing, and induce cytokine release [6].

Among all HSPs, HSP90, an ATP-dependent protein, has received widespread attention in cancer research because of its important role in carcinogenesis [25]. HSP90 also regulates human immunodeficiency virus (HIV-1) reactivation by mitogen-activated protein kinase/extracellular signal-regulated kinase and Protein Kinase C (PKC) pathways (MAPK/ERK/PKC) to influence replication and gene expression of HIV-1 virus [1, 42]. Although HSP90 has been targeted for anticancer therapy in the past decades, little research has been carried out on this protein in the protozoan parasites, a diverse group of unicellular organisms that affect more than 500 million people in the world [15, 35, 44]. It has been described in the last few years in a handful of apicomplexan parasites such as *Plasmodium falciparum* [3], *Babesia gibsoni* [53], *Theileria annulata* [34], *Eimeria tenella* [38], and *T. gondii* [2, 13]. The role of Hsp90 in growth and stage conversions between tachyzoites and bradyzoites had been described using geldanamycin (GA), but gene deletion and complementation were not involved in the research [13]. The main purpose of the present study was to illustrate the cellular functions of HSP90 in *T. gondii.* A knockout of the *Hsp90* gene was generated. We showed that *T. gondii* HSP90 was involved in bradyzoite development, played an important role in invasion and growth of the parasite *in vitro*, and was associated with virulence in BALB/c mice *in vivo*.

## Materials and methods

### Ethics

All mice were treated in strict accordance with the recommendations of the Guide for the conduct of studies with experimental animals of the People’s Republic of China. The use of animals was approved by Zhejiang University Experimental Animal Ethics Committee (Permit Number: ZJU201308-1-10-072). All efforts were made to minimize numbers and to reduce suffering of the animals used.

### Host cell and parasite cultures

The *T. gondii* RH*Δku80* strain was maintained on monolayers of African green monkey kidney (Vero) cells at 37 °C and 5% CO_2_. The culture medium was Dulbecco’s Modified Eagle’s Medium (DMEM, HyClone) supplemented with 10% fetal calf serum (FCS, HyClone), 2 mM L-glutamine, and 100 units penicillin/100 mg streptomycin [55].

### Generation of *Hsp90* gene knockout parasite

The parental strain used to generate the knockout strain was RH*Δku80*. Deletion of the *Ku80* gene involved in DNA repair via the non-homologous and joining pathway results in greater levels of homologous recombination, allowing for incorporation of reporter proteins into the 3′ end of the endogenous genes [43]. The knockout vector construct was engineered with the selectable marker *ble*, resistant to phleomycin using the pBluescript SK (+) [33]. RH*Δku80* genomic DNA was isolated using the TIANGEN genomic DNA isolation kit by following the manufacturer’s protocol (TIANGEN, Beijing). Briefly, ~1800 bp of the 5′ flanking and 3′ flanking sequences of the *Tg*Hsp90 (GenBank ID 288380) gene were amplified using the RH*Δku80* genomic DNA as templates by polymerase chain reaction (PCR). The primers were P1/P2 and P3/P4, listed in Table 1. Then, the products were flanked with KpnI and HindIII, SpeI and NotI (TAKARA, Dalian) and cloned into the pBluescript SK (+) (Promega, USA) vector. After linearization with NotI, 25–50 μg of the plasmid was electroporated into the RH*Δku80* strain by a Bio-Rad electroporation system; the electroporation conditions were 1.5 kV, 0.5 ms for three times. Transfected parasites were selected with phleomycin (Sigma, USA), high-dose extracellular treatment (50 μg/mL), and low-dose treatment (5 μg/mL) during growth in Vero cells. After a second round of selection, the individual clones were obtained by limiting dilution. The positive clones were confirmed by PCR using primers P5/P6 and P7/P8, listed in Table 1, as well as determined by Western blotting analysis.

**Table 1.** Primers used in this study.

Primers	Sequences
P1	5′-GGTACCAGAGGCCTTCAGCTTCGCGGAGA-3′
P2	5′-AAGCTTCTTGTCTCGAGCGAGGAGAGTT-3′
P3	5′-ACTAGTGCAGCTTCCAATGTCACCCG-3′
P4	5′-GCGGCCGCTGTCAAATACGAAGTTCAGCCTCTC-3′
P5	5′-CAGAGAGCAGCGCAGAGAGAACGG-3′
P6	5′-TTAAGAGATGCCTGCAAGCAATTCG-3′
P7	5′-ATGCATGACCAAGCGACGCCCAAC-3′
P8	5′-GGTGACGCTTCTCGCTTTCGCT-3′
P9	5′-CATATGGCGGACACCGAGACCTTC-3′
P10	5′-AAGCTTGTCGACCTCCTCCATCTTCGAGGT-3′
Actin-F	5′-CACGAGAGAGGATACGGCTTCACCA-3′
Actin-R	5′-CCATCGGGCAATTCATAGGACTTCTC-3′
B1-F	5′-GGAACTGCATCCGTTCATGAG-3′
B1-R	5′-TCTTTAAAGCGTTCGTGGTC-3′

### Generation of *Hsp90* complemented parasites

To further confirm the role of *Tg*Hsp90, the *Tg*Hsp90 gene was reintroduced into the *ΔHsp90* knockout parasites. The HSP90 sequence (GenBank ID AY344115.1) was amplified by PCR using primers P9/P10. Afterwards, surface antigen 1 (SAG1) promoter and granule 1 (GRA1) poly A signal sequences were added to the 5′ and 3′ end, respectively. The construct was then cloned into the pTCY vector. The vector was kindly offered by Professor Liuqun of the China Agricultural University College of Veterinary Medicine. The vector contained the chloramphenicol resistance gene *CAT*. We transformed the product to *Escherichia coli* (top10) cells and the plasmids were extracted by plasmid extraction kit (Axygen, America); 20–50 μg of the plasmid was linearized with *Kpn*I and transfected into the *ΔHsp90* parasite. Stable clones were selected by 20 μg/mL chloramphenicol (Sigma, USA). HSP90 expression was confirmed by Western blotting.

### Preparation of anti-r*Tg*Hsp90 polyclonal antibody and Western blotting

The coding region of *Tg*Hsp90 was cloned into the pMD-18T vector followed by cloning into the pET-28a vector and transfection into the *Escherichia coli* BL21 (DE3) strain (TAKARA, Dalian) for expression. The recombination protein was purified by affinity chromatography using Ni-IDA agarose (QIAGEN, Germany) and then assessed by sodium dodecyl sulfate-polyacrylamide gel electrophoresis (SDS-PAGE). The concentration of the protein was measured by the bicinchoninic acid (BCA) protein assay kit (Beyotime, Shanghai). The protein was subcutaneously injected into the test rabbits. For the first immunization, the protein concentration was 0.5 mg/kg emulsified with Freund’s complete adjuvant. The second and third immunizations were performed every two weeks; the protein concentration was 0.25 mg/kg emulsified with Freund’s incomplete adjuvant. Anti-*Tg*Hsp90 antibodies were obtained from immunized rabbits, and the titers were determined by enzyme-linked immunosorbent assay (ELISA) using recombination antigen at 13 days after the last immunization.

The parasites (10^6^) were collected and centrifuged at 3000× g for 5 min; the pellet was resuspended in cold phosphate-buffered saline (PBS) and passed three times through a 30-gauge needle syringe [14]. Purified parasites (10^6^–10^7^) were lysed with radioimmunoprecipitation assay (RIPA) buffer (Beyotime) in the presence of 1 mM phenylmethanesulfonyl fluoride (PMSF) as a protease inhibitor (Beyotime). Total proteins were quantified by the BCA protein assay kit (Beyotime); 15–20 μg of total proteins was separated on 12% SDS-PAGE gels and transferred to nitrocellulose membranes (Axygen, USA) for analysis. Rabbit anti-*Tg*Hsp90 antibody, diluted 1:1000 in 5% skim milk, which was dissolved in Tris-buffered saline with Tween 20 (TBST) (TBS buffer supplied with 0.05% Tween 20), was used to detect Hsp90. The nitrocellulose (NC) filter membrane was incubated for 2 h at 37 °C in an incubator. Horseradish peroxidase (HRP)-conjugated goat anti-rabbit immunoglobulin G (IgG), diluted 1:5000 in 5% slim milk/TBST, was used as the secondary antibody. Finally, the signals were detected by electrochemiluminescence (ECL) (CYANAGEN, Italy).

### Real-time PCR

Many stressful conditions have been connected with the development of the *T. gondii* bradyzoite. One such condition is an alkaline environment (pH 8.0–8.2), which is commonly applied in studies on the *in vitro* differentiation of *T. gondii.* Heat shock is also often used [16]. We detected the expression level of Hsp90 in order to analyze whether *T. gondii* HSP90 played a role when bradyzoites were induced under stress by alkaline pH or heat shock. Then, *T. gondii* bradyzoite genes BAG1 and MAG1 were analyzed by real-time PCR among RH*Δku80*, *ΔHsp90*, and *Hsp90*-complementation strains to further test whether *Hsp90* plays a role during bradyzoite differentiation. The relative expression level of *Hsp90* transcripts was determined by real-time RT-PCR using actin transcripts as the internal control. Primers for *T. gondii* actin are shown in Table 1; the values were calculated by the 2^-△△CT^ method.

### Immunofluorescence and invasion assays

To test the invasion of host cells, two-color (red/green) invasion assays were performed, as previously described [5]. Red/green invasion assays were performed as described for indirect immunofluorescence. RH*ku80*, *ΔHsp90*, and complemented parasites were seeded on monolayers of Vero cells on coverslips in 12-well plates with 1 × 10^5^/well. After 1, 2, and 4 h, the coverslips were washed with sterile PBS, and then fixed with 4% formaldehyde for 15 min, and external (attached) parasites were stained with rabbit antibody (SAG3), followed by washing. Monolayers were permeabilized with 0.3% Triton X-100, and internal (invaded) parasites were incubated with mouse mAb 4D-5 (SAG3), a membrane surface antigen of the SAG family in *T. gondii* generated in the laboratory. Its specificity was confirmed by specific reaction to positive porcine serum for *T. gondii*, but not to porcine serum of the animals infected with *Cryptosporidium suis*, *Mycoplasma suis*, *Streptococcus suis*, *Salmonella choleraesuis*, *Cysticercus cellulosae*, *Isospora suis*, or *Trichinella spiralis* kept in our laboratory (data not shown). Secondary antibodies were Alexa Fluor 488 (Green) and Alexa Fluor 568 (Red) (Life Technologies, USA), 4′,6-diamidino-2-phenylindole (DAPI) was added to the secondary antibody solution to stain host nuclei. Finally, the images were obtained using an Olympus confocal microscope (IX71) with 60× magnification and the numbers of intracellular parasites (green), extracellular parasites (red), and host-cell nuclei (blue) were counted. Mean values of three independent experiments were determined.

### Intracellular growth

Equal numbers (10^5^) of RH*Δku80*, *ΔHsp90*, and complemented parasites were used to infect monolayers of Vero cells grown on six-well plates; the infection ratio was 1:1. The noninvasive tachyzoites were removed after infection for 2 h and fresh medium was added to the cells. Growth of the parasites was then observed by inverted microscope. Next, 24, 48, 72, and 96 h post-infection (PI), the parasites were collected and genomic DNA was extracted using the TIANGEN genomic DNA isolation kit by following the manufacturer’s protocol (TIANGEN, Beijing). The standard curve was obtained by the known concentration of the RH*Δku80* parasites via SYBR-green real-time PCR using B1 gene primers, and the parasite numbers were calculated by interpolation from this standard curve [8]. Triplicates were used for each time point of three independent experiments.

### *In vivo* virulence of *ΔHsp90* knockout in mouse

Female BALB/c mice 6–8 weeks of age were infected intraperitoneally (IP) with 10^3^ cells of parental RH*Δku80*, *ΔHsp90* knockout, or complemented parasites. In each experiment, 12 mice were used for each type of parasite. The mice were monitored daily for survival until 28 days PI when experiments ended. This experiment was repeated three times. Survival rates were calculated from all three independent experiments.

The tissues of the liver, spleen, lungs, and brain were collected from the mice that showed clinical symptoms but had not died. We collected the organs using sterile scissors and divided them into masses of equal quality of 0.5 mg each. Genomic DNA was extracted from each tissue using a genomic DNA extraction kit (TIANGEN, Beijing). *T. gondii* DNA was detected by SYBR-green real-time PCR using B1 primer pairs (Table 1). The standard curve was obtained by the known concentration of the RH*Δku80* parasites with the primers (B1), and the parasite number was calculated by interpolation from this standard curve [7]. The results were based on three independent experiments.

### 2.10 Statistical analysis

A Student’s *t*-test was performed by SPASS (Statistical Analysis System, Version 16.0). It was considered statistically significant if *p* ≤ 0.05.

## Results

### Generating *Tg*Hsp90 knockout of *Toxoplasma gondii*

**Figure 1.**
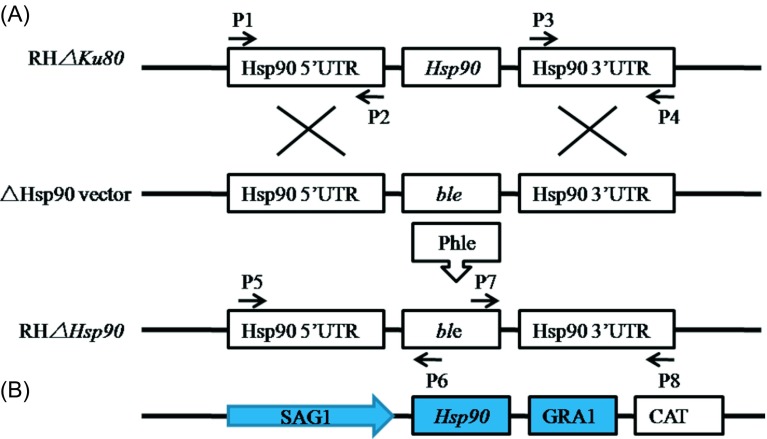
Generation of *Tg*Hsp90 knockout (*ΔHsp90*) and complementation parasites. (A) Schematic illustration of the strategy used to generate the *Tg*Hsp90 knockouts by homologous recombination. The Hsp90 coding region was replaced by the selectable marker *Ble*; the knockout vector was transfected into the RH*Δku80* strain and selected by phleomycin. (B) Diagram of genetic complementation of Hsp90. An *Hsp90* coding sequence and the *CAT* selection marker, which was flanked by *T. gondii* Tublin promoter and 3′ poly A signal (provided by Professor Liuqun, College of Veterinary Medicine, China Agricultural University), was transfected into the *Tg*Hsp90 defective strain to generate the complemented strain.

**Figure 2.**
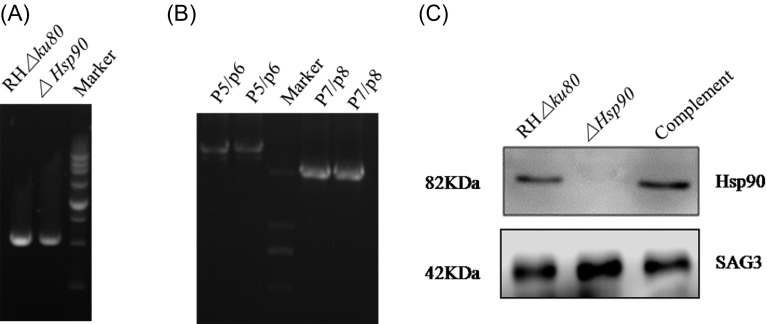
Confirmation of *ΔHsp90* and complemented parasites. (A, B) PCR analysis of *ΔHsp90* strains. The positions of the primers are shown in Figure 1A. P5/P6 and P7/P8 were used to amplify the conjunct regions of 5′ and 3′ integration of the *Ble* gene construct into the corresponding Hsp90 locus, respectively. (C) Western blotting analysis showing detection of Hsp90 in wild-type *T. gondii* RH*Δku80* and in the complemented strain, but absence in *ΔHsp90* parasites. Surface antigen (SAG3) was used as a loading control.

To understand the biological function of *Tg*Hsp90, the Hsp90 deletion vector was transfected into RH*Δku80* parasites of *T. gondii*, which was used as a wild-type strain as far as *Tg*Hsp90 was concerned. The *Tg*Hsp90 gene was replaced by the coding sequence of the *Ble* selectable marker gene under the control of the SAG1 promoter. Figure 1A shows a schematic diagram of the targeting construct *Hsp90*, designated as *ΔHsp90.* To identify the knockout clones, PCR analysis was performed using primers (P5/P6 in Table 1) from the 5′ flanking genomic regions combined with the *ble* gene and the *ble* gene combined with 3′ flanking genomic regions (P7/P8 in Table 1). The amplification of the fragment confirmed that proper integration was obtained. As shown in Figure 2A, a 529-bp fragment DNA product was successfully amplified from both *ΔHsp90* and RH*Δku80* parasites of *T. gondii*. Figure 2B shows the PCR results which confirmed that successful deficient parasite was achieved. Western blotting analysis was performed using anti-*Hsp90* rabbit antiserum (1:1000), the *ΔHsp90* knockout parasite had no detectable level of Hsp90 proteins. In contrast, the parental RH*Δku80* cells had a high level of expression of the protein (Fig. 2C).

We then generated complemental parasites of *ΔHsp90* knockout for *Hsp90* gene complementation (Figure 1B). Individual clones were selected by 20 μM chloramphenicol. The expression of HSP90 was detected by Western blotting with anti-*Hsp90* rabbit antiserum (1:1000), showing that the complemental parasites were successfully generated (Fig. 2C).

### High expression of *T. gondii* Hsp90 during bradyzoite differentiation *in vitro*

**Figure 3.**
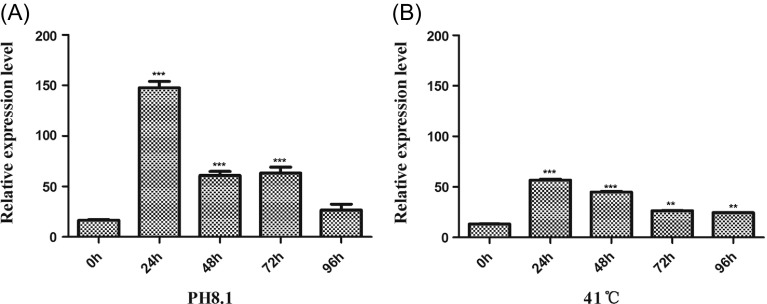
Real-time RT-PCR of Hsp90 transcriptional level during bradyzoite differentiation *in vitro*. The RH*Δku80 T. gondii* cells were exposed to alkaline treatment (pH 8.1) (A) or heat shock (41 °C) (B) for 0, 24, 48, 72 or 96 h. Total RNA was purified from the bradyzoites collected at the end of each time point. The expression levels were determined by real-time RT-PCR using actin as an internal control. Samples collected at 24, 48, 72 or 96 h were compared to those at time 0 h. “***” indicate a statistically significant difference (*p* < 0.001). Values represent mean ± *SD*, *n* = 3 experiments.

**Figure 4.**
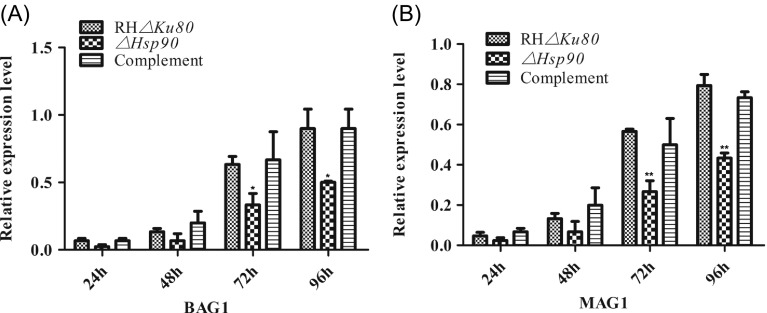
The relative expression level of BAG1 and MAG1. *ΔHsp90*, RH*Δku80* and complemented parasites (10^5^) were cultured in Vero cells for 24, 48, 72 and 96 h. The RNA was extracted by the Trizol dissociation method and detected by relative SYBR-green real-time PCR using actin transcripts as an internal control. *p < 0.05, **p < 0.01. Data represent mean ± *SD*, *n* = 3 experiments.

To determine the relative expression level of Hsp90 in *in vitro* differentiation of *T. gondii*, the RH*Δku80* parasites were exposed to alkaline pH (pH 8.1, 0.03% CO_2_) or heat shock (41 °C). The results showed that both alkaline pH and heat shock induced increasing expression of HSP90. Parasites exposed to alkaline pH for 24, 48, and 72 h increased the Hsp90 transcript level by 8.9-, 3.7-, and 3.8-fold in comparison with cells that were not exposed to such conditions at time 0 h (*p* < 0.001). Similar results were obtained for cells being exposed to heat shock (Fig. 3). Then, the relative expression level of bradyzoite genes BAG1 and MAG1 was detected by SYBR-green real-time PCR from Vero cells infected with RH*Δku80*, *ΔHsp90*, and complementary strains. The transcript level of BAG1 (*p* < 0.05) and MAG1 (*p* < 0.01) was significantly decreased when the *Tg*Hsp90 gene was lacking, compared to the RH*Δku80* parasite (Fig. 4). These data unequivocally showed that *T. gondii* HSP90 plays a role when bradyzoites are under stress induced by alkaline pH or heat shock.

### Hsp90 knockout parasites are defective in invasion of host cells

**Figure 5.**
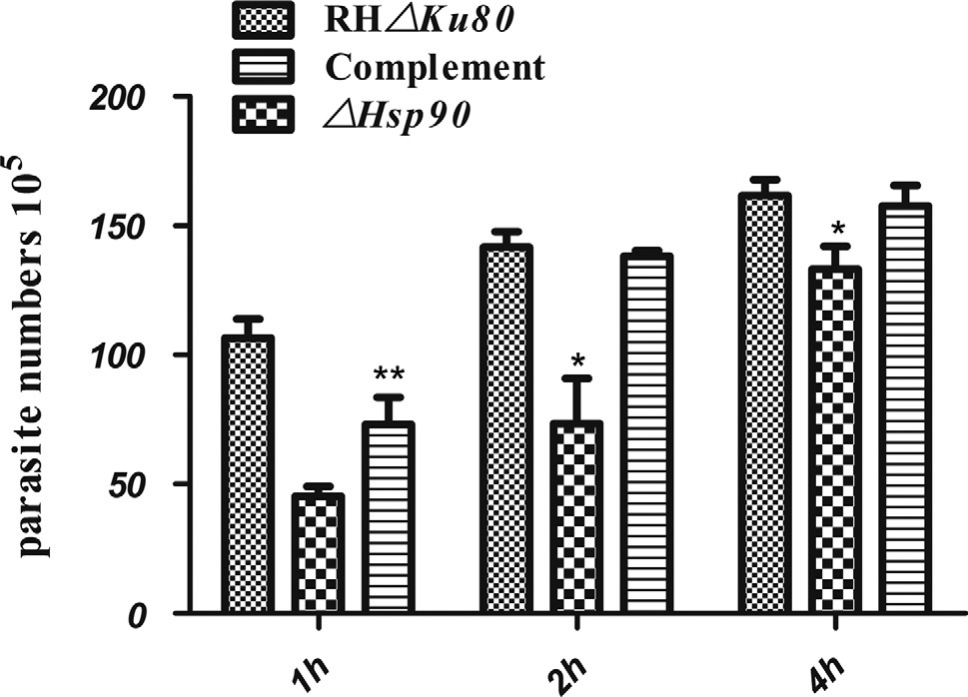
Invasion of the *ΔHsp90* parasite. The invasion ratio of *ΔHsp90*, RH*Δku80* and complemented parasites was evaluated, the points of time were 1, 2 and 4 h, and anti-SAG3 monoclonal antibody was used as the red-green attachment assay. Fields were randomly selected and data were mean ± *SD* of three independent experiments. “**” and “*” indicate statistically significant difference at *p* < 0.01 and *p* < 0.05, respectively.

We subsequently tested whether HSP90 plays a role in host-cell invasion. We used red/green antibody assays to distinguish extracellular parasites from intracellular ones. For the invasion assay, the parasites were incubated with Vero cells for 1, 2, or 4 h. Afterwards, invasion was detected by the two-color (red/green) antibody test as mentioned above. Compared to that of the RH*Δku80* parental parasite, the invasion rate of the *ΔHSP90* knockout in Vero cells was reduced by 57.5% (*p* < 0.01), 48.2% (*p* < 0.05), and 17.5% (*p* < 0.05) for the three periods of time (1, 2, and 4 h), respectively. In contrast, the rate of the knockout being complemented with *Tg*HSP90 was at a level that was similar to that of parental cells (Fig. 5). The invasion ratio assay showed that deletion of Hsp90 caused an early invasion defect of *T. gondii*, and this defect was restored by complementation of the Hsp90 gene locus. Taken together, these data showed that *Tg*HSP90 plays an important role in host-cell invasion.

### Hsp90 deletion results in a defective growth phenotype

**Figure 6.**
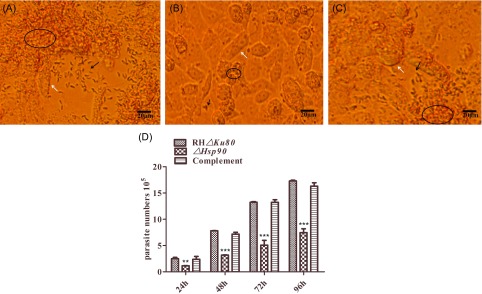
Growth of Hsp90 knockout parasite (*ΔHsp90*) *in vitro.* The parasites were cultured in African green monkey kidney (Vero) cells, 10^5^ *T. gondii* were added to the 6-well plates, and the infection ratio was 1:1. Observation of RH*Δku80* (A), *ΔHsp90* (B), and complemented (C) parasites by inverted microscope. The plaque produced by *ΔPKAR* strains (Fig. 6B) was significantly smaller than that of RH*Δku80* and complemented parasites (Figs. 6A, 6C), scale bar = 20 μm. *T. gondii* tachyzoites and Vero cells were indicated by arrows, *T. gondii* (black arrow), Vero cells (white arrow). (D) The parasites were collected at the same time, and genomic DNA was extracted by TIANGEN kit. *T. gondii* DNA was detected by SYBR-green real-time PCR using B1 primer pairs, the standard curve was obtained by the known concentration of the RH*Δku80* parasites with the primers (B1), and the parasite number was calculated by interpolation from this standard curve. **p < 0.01, ***p < 0.001.

To evaluate the growth rate of Hsp90 knockout, *ΔHsp90*, RH*Δku80*, and complemented parasites were added to monolayer Vero cells at a 1:1 ratio. The parasite plaques were examined using an inverted microscope, and the results showed that the plaque produced by *ΔPKAR* strains (Fig. 6B) was significantly smaller than that of RH*Δku80* and complemented parasites (Figs. 6A, 6C). At 24, 48, 72, and 96 h of incubation, cell cultures were collected and parasite numbers were determined with SYBR-green real-time PCR that amplified specific oligonucleotides of the B1 gene of *T. gondii*. As shown in Figure 6D, the growth of *ΔHsp90* knockout decreased by 58.1%, 40.6%, 38.3%, and 43.1% at the time points 24, 48, 72, and 96 h, respectively, in comparison with that of the RH*Δku80* parental cells. The difference was statistically significant at 24 h (*p* < 0.001). In contrast, the growth of the *ΔHsp90* knockout complemented with the HSP90 gene was comparable to that of the RH*Δku80* parental cells (*p* > 0.05). These data collectively demonstrated that Hsp90 was required for the normal growth of *T. gondii*.

### *Tg*Hsp90 is essential for virulence in the RH strain

**Figure 7.**
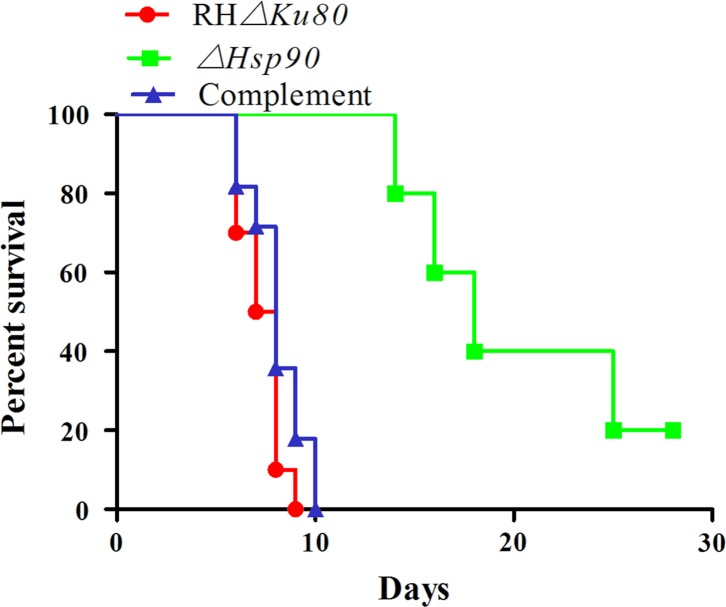
Virulence of *Hsp90* knockouts in BALB/c mice Purified tachyzoites from different strains were intraperitoneally injected (10^3^ parasites/mice) into 6–8 weeks of female BALB/c mice, 10 mice/group; the survival rate was monitored. The experiment was performed three times.

The above observation of reduced growth *in vitro* was intriguing. We next investigated whether Hsp90 contributed to virulence of the RH strain *in vivo*. Equal numbers of *ΔHsp90*, RH*Δku80*, and complemented parasites were injected IP to BALB/c mice at a dose of 10^3^ parasites/mouse. The mice that received the wild-type parasites developed clinical signs at day 4 PI, and all died between 6 and 9 days PI. In contrast, the mice given the *ΔHsp90* knockout cells showed no clinical signs before 14 days PI, and about a quarter of the mice remained alive even until 28 days PI when the experiment ended (Fig. 7). These results clearly showed that deletion of the *Tg*HSP90 gene attenuated *T. gondii* and greatly reduced parasite virulence in mice.

**Figure 8.**
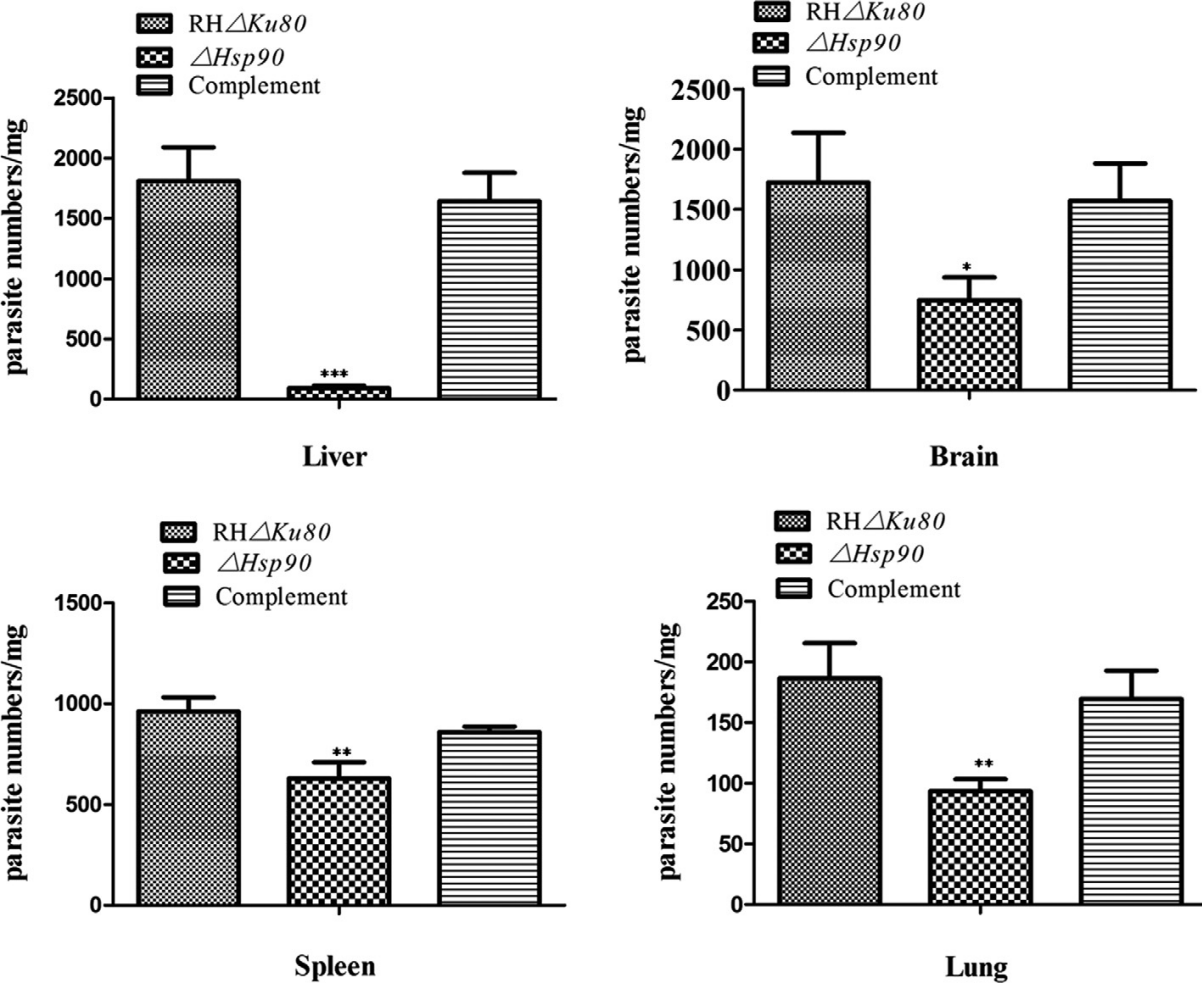
Analysis of parasite burden in BALB/c mice. The parasite burden was also detected in infected mice when the symptoms of *T. gondii* infection appeared. Parasite burden was detected in liver, brain, spleen, and lung tissues. The data were means ± *SD* for three experiments. *p < 0.05, **p < 0.01, ***p < 0.001.

We also determined the parasite load in tissues of *T. gondii* infected mice in the liver, spleen, lungs, and brain. The tissues were collected from the animals at the same time point when they were euthanized. SYBR-green real-time PCR was used to quantify parasite loads in each tissue. Results revealed that all tissues were *T. gondii* infection positive, but the most numerous parasites were observed in the brain and liver, which was consistent with other research [9]. Specifically, the parasite load in the liver of mice infected with RH*Δku80* parental cells was 1809/mg, whereas that of *ΔHsp90* knockout mice was 91.2, which was a 19.8× reduction (*p* < 0.001). The average parasite load in the brain was reduced by 3.2-fold (*p* < 0.05). Similarly, the parasite loads in the spleen and lungs were also significantly lower in mice receiving knockout parasites than parental cells (*p* < 0.01) (Fig. 8). In contrast, the mice having received the complemented parasites had similar parasite loads to the animals that were infected with parental cells in all four kinds of tissues, i.e. the liver, brain, spleen, and lungs (Fig. 8). Taken together, the results of *in vivo* infection in a mouse model clearly showed that deletion of the HSP90 gene attenuated *T. gondii* greatly, as demonstrated by a much longer survival period and a high magnitude reduction in parasite load.

## Discussion

Hsp90 proteins of apicomplexan protozoa play a pivotal role in the parasite’s biology and virulence. *Plasmodium* Hsp90 had been shown to regulate parasite growth in human erythrocytes [52]. Geldanamycin (GA) competitively binds to the N-terminal ATP-binding pocket of Hsp90, leading to the inhibition of its chaperone cycle and reducing the capacity of *E. tenella* to invade and grow in host cells [38]. Exposure of *Babesia gibsoni* to heat shock at 43 °C induced a dramatic increase in its Hsp90 transcripts [53]. Hsp90 is important in other protozoa as well. In *Leishmania donovani*, Hsp90 is involved in the maintenance of motility and proliferation, and serves as part of the signal transduction pathways that regulates stage-specific gene expression [20, 21, 36]. Interestingly, in closely related *Trypanosoma cruzi*, Hsp90 was related to response to stress but not stage differentiation [18].

Although the *T. gondii* Hsp90 gene has been described [2, 13], there is very limited information available regarding its biological function. In the current studies, we demonstrated that *T. gondii* Hsp90 plays an important role in bradyzoite differentiation, host-cell invasion, and growth and virulence.

*T. gondii* interconverts between rapidly dividing tachyzoites and latent encysted bradyzoites. The encysted bradyzoites are critical for the maintenance of *T. gondii* in nature, especially for transmitting the parasite among various intermediate hosts. The interconversion process is accompanied by morphological changes and metabolic adaptations [11, 37]. Toursel and colleagues confirmed that a gene encoding Hsp60 of *T. gondii* was selectively expressed during the intracellular development of the parasite [49]. The regulatory regions of the *T. gondii* Hsp70 gene were analyzed by a *β*-galactosidase expression vector transfected into the RH strain. At pH 8.1 (pH stress), which leads to bradyzoite development, the expression level of Hsp70 was increased [32]. However, the factors and mechanisms governing the interconversion are poorly understood at the molecular level. Studies investigating the switch between the tachyzoite and bradyzoite have confirmed that the SAG1-related sequence (SRS) [27], zinc finger protein (ZFP1) [51], cyclic AMP-dependent protein kinase subunit 3 (PKAc3) [48], mitogen-activated protein kinase 1 (MAPK1) [7], and autophagy-related proteins (Atg) [29] are all involved in bradyzoite differentiation. In the present study, the transcriptional level of the *Hsp90* gene was determined when *T. gondii* cells were under stress at 41 °C or pH 8.1. It was found that *Hsp90* transcripts increased under these stressful conditions, which was similar to the findings of research carried out by Echeverria et al. [13]. The results indicated that developmental differentiation of *T. gondii* may be governed by both exogenous stress factors and complex endogenous cellular environments.

Recent studies have indicated that Hsp90 is crucial for the survival and growth of parasites. Cellular components involved in these processes include up-regulation in transporters, cysteine proteases, cytoskeletal proteins, and components of the proteasome, just to name a few [10, 47]. In the *Leishmania donovani* parasite, the co-chaperone of Hsp90 called P23 acts against various stresses, especially the inhibitory effect of GA, and protects the cells from the harmful effect of the Hsp90 inhibitor, and as a result, the cells grow normally even under such conditions [22]. Other studies of *Leishmania donovani* have indicated that the proliferation, viability, and infectivity of the kinetoplastid parasite were affected by Hsp90-Sti1 interaction. Sti1 is a binding motif of Hsp90. The interaction compound is involved in stage-specific phosphorylation, signal transduction cascades, and the chaperone phosphorylation protein modifications related to parasite viability [21, 36]. In the present study, we demonstrated that deletion of Hsp90 in *T. gondii* made the parasites grow slower in Vero cells than the wild-type parasite RH*Δku80*, which indicated that Hsp90 participated in cellular growth, and this process may be accomplished by its interaction with co-chaperones.

In a number of organisms, calcineurin, a calcium-activated protein phosphatase, is important for regulating responses to stresses [47]. It has been shown in murine models that calcineurin is an important virulence factor [4]. Interestingly, *S. cerevisiae* Hsp90 interacts with calcineurin and Hsp90 increases calcineurin activity in a dose-dependent manner, which may affect the growth and survival of cells [24]. In the current study, we showed that mice receiving intraperitoneal injections of *ΔHsp90* lived much longer than the mice inoculated with either RH*Δku80* or complemental parasites*.* Our data further demonstrated lower parasite loads in tissues of the liver, lungs, spleen, and brain in mice given HSP90 knockout parasites, and that the heaviest parasite loads were found in the liver and brain, which was consistent with a previous report [9].

Previous studies have shown that the heat shock response can cause the dissociation of the IκB kinase (IKK) complex which is the major activator of the NF-κB complex, then inhibits the activation of the NF-kB signaling pathway [45], and inhibits innate immunity or cellular responses in order to help cells to survive tissue injury [40]. Consequently, the function of *Tg*HSP90 in the growth of *T. gondii* may involve multiple cellular signaling pathways. Future studies of *Tg*HSP90 will be needed to clarify the interaction between HSP90 and the signaling molecules. The *T. gondii* HSPs are divided into six major families that are grouped according to their molecular weight: small heat shock proteins, Hsp40, Hsp60, Hsp70, Hsp90, and Hsp100. They have significant homologies, suggesting that other heat shock proteins may play an important role in the survival of the Hsp90 knockout parasites, but the specific mechanisms were unknown, and this needs further study.

In conclusion, the present study constructed *Toxoplasma gondii* Hsp90 (*Tg*Hsp90) knockout and complementation strains. Our results show that TgHsp90 contributes to bradyzoite development, invasion, and replication of *T. gondii* in the host cell, and has an important effect on parasite virulence *in vivo*. These data will provide a useful basis for further study of the gene function of *Tg*Hsp90 and for screening drug targets of *T. gondii*.

## Conflict of interest

The authors have no conflict of interest.
